# Inclusion bodies: not that bad…

**DOI:** 10.3389/fmicb.2014.00056

**Published:** 2014-02-14

**Authors:** Ana Ramón, Mario Señorale-Pose, Mónica Marín

**Affiliations:** Sección Bioquímica, Facultad de Ciencias, Universidad de la RepúblicaMontevideo, Uruguay

**Keywords:** protein aggregation, bacterial inclusion bodies, protein folding, recombinant protein expression, conformational disease model, drug delivery systems, nanoparticules

## Abstract

The formation of inclusion bodies (IBs) constitute a frequent event during the production of heterologous proteins in bacterial hosts. Although the mechanisms leading to their formation are not completely understood, empirical data have been exploited trying to predict the aggregation propensity of specific proteins while a great number of strategies have been developed to avoid the generation of IBs. However, in many cases, the formation of such aggregates can be considered an advantage for basic research as for protein production. In this review, we focus on this positive side of IBs formation in bacteria. We present a compilation on recent advances on the understanding of IBs formation and their utilization as a model to understand protein aggregation and to explore strategies to control this process. We include recent information about their composition and structure, their use as an attractive approach to produce low cost proteins and other promising applications in Biomedicine.

## Introduction

The deciphering of the genetic code and the availability of the first tools and basic procedures in genetic engineering opened the way for protein production in bacteria. This apparently easy goal encountered an unexpected difficulty: the accumulation of the protein of interest as insoluble form and the generation of inclusion bodies (IBs). Since then the generation of IBs has been traditionally considered an obstacle to avoid and difficult to predict. Notwithstanding in the last decade, IBs have been observed from a different perspective and their study gained considerable interest. From this point of view, the formation of IBs in bacteria is seen as part of a general cellular response related to the presence in the cell of unfolded proteins and as a pathway for the control of aggregation. From this perspective, IBs constitute a valuable model to better understand protein aggregation in eukaryotes, and for the search of specific inhibitors or disaggregation approaches, in relation to relevant conformational diseases. On the other hand, the production of proteins as aggregates in IBs opened new interesting perspectives for diverse applications in Biomedicine. They can be an almost pure source of recombinant protein, and because of their particular structural and functional characteristics they can be potentially exploited as naturally immobilized enzymes or as nanomaterials. In this minireview, we focus on this positive side of IBs formation in bacteria. We include recent information about their composition, formation and structure and their use as an attractive approach to produce proteins at low cost. We also review the role of IBs as a model to understand protein aggregation and to explore strategies to control this process. Finally we describe some promising applications of bacterial IBs as systems for controlled drug delivery and nanotechnology applications.

## Protein aggregation as a conserved cellular response

Diverse conditions can lead to an alteration of protein homeostasis and to protein aggregation in all living cells. Among others, the presence of unfolded proteins is increased after a heat shock or other environmental stress conditions. Some mutations can lead to the synthesis of unstable protein structures, as well. Another current event is provided by fast and high expression of recombinant proteins in bacteria, leading to the formation of IBs. Recent evidence indicates that in the cell, the quality control system—regulating chaperones and proteases levels—and protein aggregation and disaggregation are part of a cellular response to altered protein homeostasis. In a recent review Tyedmers et al. (2010) described protein aggregation as a regulated process in bacteria, yeast and mammalian cells. Interestingly, despite differences, similarities of protein aggregation in these cells reflect the universality of the response. In particular, the subcellular localization of aggregates is not random but well-defined in each cellular type. The IBs in bacteria are mainly localized in the cell poles, and also in septation sites, whereas in yeast protein deposits are close to vacuoles or to the nucleus. In mammals deposits in aggresomes are associated to the nucleolus. Another relevant observation is the similarity at the level of protein structure between bacterial IBs and the amyloid aggregates characteristic of several human diseases, the so-called “conformational diseases”, in which protein deposits are observed in specific tissues or cells. This is the case for several neurodegenerative diseases such as Alzheimer or Parkinson, among others.

Under a heat shock stress, about 150–200 different proteins were identified as aggregation-prone in *Escherichia coli* (Winkler et al., 2010). The formation of aggregates can be reversed by the combined effect of chaperones and protease activities and the disaggregation machinery. As mentioned, aggregates are mainly located at cellular poles by mechanisms not yet completely understood. Recently Winkler et al. proposed that nucleoid occlusion is the main driving force which determines the number and positioning of the protein aggregates in *E. coli*. Also, authors argued against the idea that an active targeting mechanism was involved in polar localization. Interestingly, polar localization allows an asymmetric partitioning of protein aggregates between daughter cells. This asymmetry allows an increased cell division rate in the population devoid of aggregates, beneficial for the ageing of the bacterial cell population (Winkler et al., 2010).

## Prediction of a protein's aggregation propensity

The aggregation behavior of a protein is strongly determined by intrinsic properties of its amino acidic sequence. This observation supported the development of computational methods to predict protein aggregation propensity. Interestingly, recent algorithms take into account not only the primary sequence of the polypeptide, but also experimental proteome data, including information about cellular localization, cytosolic, periplasmic and membrane proteins. The analysis performed by De Groot and Ventura (2010) using AGGRESCAN -an algorithm developed by their group, (Conchillo-Sole et al., 2007; De Groot et al., 2012)-, indicates that the aggregation propensity of bacterial proteins is associated with their length, conformation, location, function, and abundance. Recently, in AMYLPRED2 11 predictive methods were considered, trying to produce a consensus prediction of amyloidogenic determinants/“aggregation-prone” peptides in proteins, from sequence alone (Tsolis et al., 2013) (http://biophysics.biol.uoa.gr/AMYLPRED2). It is worth mentioning that other factors affect the aggregation of recombinant protein expression in bacteria such as temperature and growth rate, fusion to soluble protein tags, specific codon usage, tRNA availability, and general optimization of codons in the heterologous expressed sequence (Cortazzo et al., 2002; Rosano and Ceccarelli, 2009).

## IBs structure: a sponge-like supramolecular organization

The formation and structure of IBs in *E. coli* expressing heterologous proteins has been extensively analyzed. Several excellent reviews summarize the current knowledge on IBs structure and formation (De Groot et al., 2008, 2009; Wang, 2009; Sabate et al., 2010; Garcia-Fruitos et al., 2011, 2012). Here we present a concise summary and extend a little more on the most recent published data.

IBs are normally observed in the cytoplasm of the producing bacteria as dense, large and apparently spherical or cylindrical particle, ranging from 0.2 to 1.2 μm, composed of 80–95% of the heterologous expressed protein. IBs may also contain other proteins, like small heat shock proteins (IbpA and IbpA) and chaperones (like the DnaK system), phospholipids from membranes and nucleic acids and other background proteins that co-purify with aggregates (Jurgen et al., 2010). Interestingly, the cellular composition of IBs evolves during cell growth so that cellular proteins are predominant during the first steps of formation, while heterologous proteins become predominant at the end. These changes occur in concert with the evolution of other parameters inherent to cell growth, such as division time and growth rate, leading to the idea that aging of bacterial population could be related to protein aggregation (Lindner et al., 2008).

Different approaches have revealed that IBs bare a characteristic cross-β structure, resembling that found in amyloid fibers associated to a wide variety of human degenerative diseases. Notwithstanding, IBs may also contain variable amounts of natively folded proteins or partially folded proteins that can acquire their native conformation even if they are embedded in an aggregate (Gonzalez-Montalban et al., 2008). In fact aggregates have been found to be composed of a wide spectrum of conformations, ranging from native conformation to misfolded aggregates (Schrodel et al., 2005; Rinas et al., 2007). Moreover, aggregation and disaggregation have been shown to occur simultaneously *in vivo* in actively producing recombinant bacteria (Carrio and Villaverde, 2002). The solubilized proteins can then reach their native state or alternatively suffer partial proteolytic degradation (Corchero et al., 1997; Carrio et al., 1999; Cubarsi et al., 2001; Lethanh et al., 2005; Vera et al., 2005; Rinas et al., 2007). The proportion of functional protein is characteristic of the target protein's sequence (Upadhyay et al., 2012), but also depends on cell growth temperature (De Groot and Ventura, 2006; Peternel et al., 2008) and on the genetic traits of the host strain (Garcia-Fruitos et al., 2009)

The cross-β sheet regions have been shown to be refractory to proteinase K (PK) digestion, while native or native-like structures are highly sensitive to PK digestion. Using a GFP reporter model, Cano-Garrido et al. (2013) have shown that when IBs are submitted to mild digestion with the protease their morphology or size is not affected while fluorescence emission and density are notably diminished. They propose that IBs present a sponge-like structure, where the PK resistant fibrils constitute a scaffold which confers mechanical stability to IBs, while the functional, PK sensitive fraction accumulate in the gaps of this scaffold.

In accordance to this, Walther et al. (2014) suggested a mechanism of pore diffusion out of a barrier layer for the solubilization of IBs. They propose that the solubilization process involves different layers in the IBs: a core, consisting of the IBs agglomerates, a reactive and a diffusion layer. The densely packed inner cores of protein shrink as the solubilized protein diffuses to the outer layers and subsequently through a porous barrier layer into free solution. The authors propose that this model correlates well with the IBs structure suggested by Cano-Garrido et al. (2013), the barrier layer corresponding to the amyloid scaffold, which becomes visible only as solubilization progresses.

## IBs a model to study protein aggregation related to conformational diseases

The formation and disaggregation of bacterial IBs gained growing attention as models to study insoluble protein deposits observed in some complex human diseases, as in the so-called “conformational diseases”. This approach is strongly supported by the concept that protein aggregation is part of a conserved cellular response. Three examples have been chosen to illustrate how IBs are employed as a model to study aggregation proteins involved in particular human diseases and as a useful screening approach for the search for aggregation inhibitors.

### Expanded polyQ in huntington disease

Huntington disease is a neurodegenerative disorder that affects muscle coordination, followed by cognitive and psychiatric problems. The disease is caused by mutations in the Huntingtin gene, in which expansion of the triplet CAG within the first exon of the gene produces a protein carrying stretches of repeated glutamines (polyQ). When polyQ exceeds a critical length, huntingtin protein undergoes amyloid aggregation (Orr and Zoghbi, 2007). *E. coli* has been employed to follow *in vivo* the aggregation process of an artificial protein harboring a polyglutamine (polyQ) tract (Ignatova et al., 2007). *E. coli* growth rate was found to be sensitive to the protein conformational state, and showed that misfolded peptides and soluble aggregates were cytotoxic (Miller et al., 2010).

Related to some pathologies, the relationship between “aggregation” and “toxicity” is often controversial. This question was recently explored in *E. coli* by expressing PolyQ-Containing Ataxin-3 (Invernizzi et al., 2012). For this purpose, the toxicity of three variants expressed in *E. coli* was determined according to reduction of growth rate. The authors showed that toxicity was correlated to the formation of soluble cytosolic oligomers, but not to peptide aggregation. Instead, interestingly, the aggregates appeared to be protective against cell toxicity (Invernizzi et al., 2012).

### Prion protein expression in bacteria

Prions are protein aggregates with self-perpetuating ability and thus infectious (reviewed in Villar-Pique and Ventura, 2012). Prions are involved in transmissible spongiform encephalopathies (TSEs), a family of rare progressive neurodegenerative disorders that affect both humans and animals. Bacterial IBs have been exploited as a tool for the study of the structural and functional characteristics of prions. Het-s, from the fungus *Podospora anserina*, was the first prion protein whose bacterial IBs were shown to display amyloid-like properties (Sabate et al., 2009; Wasmer et al., 2009). These *E. coli*-produced aggregates were transfected into prion-free fungal strains, and were shown to promote prionic conversion of Het-s at levels comparable to those induced by homologous amyloid fibrils (Sabate et al., 2009). A similar observation was reported in the case of the yeast prion Sup35. The IBs of this protein were used to induce the [PSI+] prion in [psi-] prion-free yeast strains. These results highlight the fact that the infectivity rate can be easily modulated by tuning the environmental conditions during the formation of IBs (Radchenko et al., 2011; Sabate et al., 2012).

### Alzheimer Aβ42 aggregation

There is an increasing interest in developing methods to identify cellular factors that trigger the aggregation of proteins inside the organism as well as to discover drugs able to interfere with these factors. Villar-Pique et al. (2012) describe a fast, cost-effective high-throughput approach to study conditions and molecules that affect Aβ 42 aggregation. The assay is based on the use of IBs formed by an Aβ 42-GFP fusion protein in bacteria. They showed the ability of the approach to detect the effect of metal ions on Aβ 42 aggregation as well as to identify compounds that block metal-induced reaction. The authors further propose that, as many proteins form IBs when expressed in bacteria (De Groot et al., 2009), this approach may have a much larger applicability in the search for aggregation modulators in conformational disorders (Villar-Pique et al., 2012).

## Methods for the recovery of functional proteins from IBs

Considering IBs as a source of almost pure proteins, one possible way is to attain the dissolution of aggregates in order to obtain native-folded, active protein. The challenge is then to solubilize and refold as much aggregated protein as possible and obtain a stable, functional product. The cost of the whole process must be taken into consideration if the aim is to produce a large-scale manufactured product.

The rate and yield of the solubilization process seem to be influenced by the conditions used, like chaotrope addition, concentration, temperature, pressure, etc. Even if there is no general method to solubilize and refold a protein and the strategy in each case must be “custom-made”, most IBs protein-recovery procedures include the following steps (Burgess, 2009; Basu et al., 2011): (i) overexpression of the selected protein in an appropriate host strain; (ii) isolation of IBs; (iii) solubilization of IBs; (iv) refolding (including disulfide bond formation when necessary); (v) high-resolution chromatography (vi) quality control of obtained material.

Solubilization and refolding are the most critical steps in the procedure and successful conditions still depend mostly on trial-and-error strategies (Burgess, 2009). Notwithstanding, efforts have been made to rationalize the refolding step, the free REFOLD database (http://refold.med.monash.edu.au; Chow et al., 2006a,b) can be a useful tool for the design of procedures for the refolding and purification of recombinant proteins.

There are very good recent reviews that compile the different methods employed in the different steps of the recovery process. In accordance to the above mentioned findings on the structure and solubilization mechanisms of IBs, the tendency is to employ milder extraction conditions, avoiding strong denaturation and refolding conditions. In Table 1 we listed the most recent approaches reported in the last 3 years.

**Table 1:** **Novel approaches employed in the recovery of proteins from IBs**.

**Recovery step**	**Strategy**	**Advantage**	**Protein**	**References**
Solubilization/Refolding	6M n-propanol	Chaotropic effect at high concentration/kosmotropic effect at dilution helps refolding	r-hGH	Singh et al., 2012
Solubilization/Refolding	Aminoacid-based detergent + arginine assisted refolding	Arg minimizes protein-protein interactions	scFvs	Kudou et al., 2011a,b
Solubilization	Non-reducing buffer + low concentration of urea	Avoids inappropriate disulfide bonds and aggregates. Suitable for proteins that aggregate with native-like structures	PrP	Walsh et al., 2012
Solubilization	Microwave assistance	Shortens solubilization time	various	Datta et al., 2013
Refolding	High hydrostatic pressure	No previous protein denaturation required	LECT2	Zheng et al., 2013
Refolding	Dialysis against PE-PEG (Polyethylene-Polyethyleneglycol)	Simple and cheap method; high yield	FhuA Δ1-159	Dworeck et al., 2011
Solubilization	Alkaline-shock	No use of chaotropic agents	adiponectin	Heiker et al., 2010
Refolding	Solid-phase refolding in cation-exchange resin with decreasing gradient of urea	Minimize time and number of steps	BMP-2 dimers	Rane et al., 2013

## IBs nanoparticules and controlled delivery of drugs

A recent report showed that peptide hormones of the pituitary gland are stored intracellularly as amyloid aggregates within the secretion granules. The amyloid cross-β structure provides a very stable and highly compacted state from which controlled release of functional monomeric hormone can take place upon signaling (Maji et al., 2009). A somewhat similar situation occurs with bacterial IBs. In fact, IBs are composed by amyloid-like aggregates from which substantial amounts of functional recombinant protein can be released *in vivo* as well as under mild (non-denaturing) conditions *in vitro*. This feature—reminiscent of a drug delivery system—led to the concept of the “nanopills,” i.e., nanoparticles which are able to release proteins with therapeutic effects directly from the inside of cells (Vazquez et al., 2012). As a proof of concept, Vazquez et al. demonstrated that the addition of different types of IBs to the culture medium of mammalian cells, for example IBs composed of HSP70, leukemia inhibitory factor or catalase, were able to rescue them from cis-platinum, serum deprivation or oxidative stress, respectively. Also, dihydrofolate reductase IBs complemented the intrinsic cell deficiency of this enzyme. Furthermore, IBs are spontaneously internalized by cultured cells, as was directly demonstrated with green fluorescent protein IBs (Villaverde et al., 2012). Following a similar approach, Liovic et al. (2012) introduced keratin 14 IBs into epithelial cells which do not normally express this protein, and found that intracellular keratin filaments start to form.

## Concluding remarks

The common view of IBs as undesirable by-products in recombinant protein production have been lastly reconsidered, in view of the potential of these structures for different purposes. On the other hand, IBs provide to constitute an easy to handle model for the study of the molecular basis of conformational diseases. On the other hand, IBs provide a source of almost pure polypeptides and are a potentially useful source of ready-to-use protein. In this sense, the aim is then to obtain IBs containing as much folded, functional protein as possible. The design of strategies to reach this aim requires a deep knowledge of IB's structure and formation, in order to identify possible molecular targets which can be “tuned” to improve the protein recovery yield or to obtain IBs with the desired characteristics to allow their use as enzyme carriers or nanomaterials.

### Conflict of interest statement

The authors declare that the research was conducted in the absence of any commercial or financial relationships that could be construed as a potential conflict of interest.

## References

[B1] Basu A. Li X. Leong S. S. (2011). Refolding of proteins from inclusion bodies: rational design and recipes. Appl. Microbiol. Biotechnol. 92, 241–251 10.1007/s00253-011-3513-y 21822901

[B2] Burgess R. R. (2009). Refolding solubilized inclusion body proteins. Methods Enzymol. 463, 259–282 10.1016/S0076-6879(09)63017-2 19892177

[B3] Cano-Garrido O. Rodriguez-Carmona E. Diez-Gil C. Vazquez E. Elizondo E. Cubarsi R. (2013). Supramolecular organization of protein-releasing functional amyloids solved in bacterial inclusion bodies. Acta Biomater. 9, 6134–6142 10.1016/j.actbio.2012.11.033 23220450

[B4] Carrio M. M. Corchero J. L. Villaverde A. (1999). Proteolytic digestion of bacterial inclusion body proteins during dynamic transition between soluble and insoluble forms. Biochim. Biophys. Acta 1434, 170–176 10.1016/S0167-4838(99)00177-6 10556571

[B5] Carrio M. M. Villaverde A. (2002). Construction and deconstruction of bacterial inclusion bodies. J. Biotechnol. 96, 3–12 10.1016/S0168-1656(02)00032-9 12142138

[B6] Chow M. K. Amin A. A. Fulton K. F. Fernando T. Kamau L. Batty C. (2006a). The REFOLD database: a tool for the optimization of protein expression and refolding. Nucleic Acids Res. 34, D207–D212 10.1093/nar/gkj080 16381847 PMC1347443

[B7] Chow M. K. Amin A. A. Fulton K. F. Whisstock J. C. Buckle A. M. Bottomley S. P. (2006b). REFOLD: an analytical database of protein refolding methods. Protein Expr. Purif. 46, 166–171 10.1016/j.pep.2005.07.022 16150607

[B8] Conchillo-Sole O. De Groot N. S. Aviles F. X. Vendrell J. Daura X. Ventura S. (2007). AGGRESCAN: a server for the prediction and evaluation of “hot spots” of aggregation in polypeptides. BMC Bioinformatics 8:65 10.1186/1471-2105-8-65 17324296 PMC1828741

[B9] Corchero J. L. Cubarsi R. Enfors S. Villaverde A. (1997). Limited *in vivo* proteolysis of aggregated proteins. Biochem. Biophys. Res. Commun. 237, 325–330 10.1006/bbrc.1997.7132 9268709

[B10] Cortazzo P. Cervenansky C. Marin M. Reiss C. Ehrlich R. Deana A. (2002). Silent mutations affect *in vivo* protein folding in *Escherichia coli*. Biochem. Biophys. Res. Commun. 293, 537–541 10.1016/S0006-291X(02)00226-7 12054634

[B11] Cubarsi R. Carrio M. M. Villaverde A. (2001). *In situ* proteolytic digestion of inclusion body polypeptides occurs as a cascade process. Biochem. Biophys. Res. Commun. 282, 436–441 10.1006/bbrc.2001.4583 11401478

[B12] Datta I. Gautam S. Gupta M. N. (2013). Microwave assisted solubilization of inclusion bodies. Sustain. Chem. Process. 1, 2 10.1186/2043-7129-1-2

[B13] De Groot N. S. Castillo V. Grana-Montes R. Ventura S. (2012). AGGRESCAN: method, application, and perspectives for drug design. Methods Mol. Biol. 819, 199–220 10.1007/978-1-61779-465-0_14 22183539

[B14] De Groot N. S. Espargaro A. Morell M. Ventura S. (2008). Studies on bacterial inclusion bodies. Future Microbiol. 3, 423–435 10.2217/17460913.3.4.423 18651814

[B15] De Groot N. S. Sabate R. Ventura S. (2009). Amyloids in bacterial inclusion bodies. Trends Biochem. Sci. 34, 408–416 10.1016/j.tibs.2009.03.009 19647433

[B16] De Groot N. S. Ventura S. (2006). Effect of temperature on protein quality in bacterial inclusion bodies. FEBS Lett. 580, 6471–6476 10.1016/j.febslet.2006.10.071 17101131

[B17] De Groot N. S. Ventura S. (2010). Protein aggregation profile of the bacterial cytosol. PLoS ONE 5:e9383 10.1371/journal.pone.0009383 20195530 PMC2828471

[B18] Dworeck T. Petri A. K. Muhammad N. Fioroni M. Schwaneberg U. (2011). FhuA deletion variant Delta1-159 overexpression in inclusion bodies and refolding with Polyethylene-Poly(ethylene glycol) diblock copolymer. Protein Expr. Purif. 77, 75–79 10.1016/j.pep.2010.12.006 21168506

[B19] Garcia-Fruitos E. Rodríguez-Carmona E. Díez-Gil C. Ferraz R. M. Vázquez E. Corchero J. L. (2009). Surface cell growth engineering assisted by a novel bacterial nanomaterial. Adv. Mater. 21, 4249–4253 10.1002/adma.200900283

[B20] Garcia-Fruitos E. Sabate R. De Groot N. S. Villaverde A. Ventura S. (2011). Biological role of bacterial inclusion bodies: a model for amyloid aggregation. FEBS J. 278, 2419–2427 10.1111/j.1742-4658.2011.08165.x 21569209

[B21] Garcia-Fruitos E. Vazquez E. Diez-Gil C. Corchero J. L. Seras-Franzoso J. Ratera I. (2012). Bacterial inclusion bodies: making gold from waste. Trends Biotechnol. 30, 65–70 10.1016/j.tibtech.2011.09.003 22037492

[B22] Gonzalez-Montalban N. Natalello A. Garcia-Fruitos E. Villaverde A. Doglia S. M. (2008). *In situ* protein folding and activation in bacterial inclusion bodies. Biotechnol. Bioeng. 100, 797–802 10.1002/bit.21797 18351678

[B23] Heiker J. T. Kloting N. Bluher M. Beck-Sickinger A. G. (2010). Access to gram scale amounts of functional globular adiponectin from *E. coli* inclusion bodies by alkaline-shock solubilization. Biochem. Biophys. Res. Commun. 398, 32–37 10.1016/j.bbrc.2010.06.020 20541528

[B24] Ignatova Z. Thakur A. K. Wetzel R. Gierasch L. M. (2007). In-cell aggregation of a polyglutamine-containing chimera is a multistep process initiated by the flanking sequence. J. Biol. Chem. 282, 36736–36743 10.1074/jbc.M703682200 17942400 PMC2892112

[B25] Invernizzi G. Aprile F. A. Natalello A. Ghisleni A. Penco A. Relini A. (2012). The relationship between aggregation and toxicity of polyglutamine-containing ataxin-3 in the intracellular environment of *Escherichia coli*. PLoS ONE 7:e51890 10.1371/journal.pone.0051890 23251648 PMC3522584

[B26] Jurgen B. Breitenstein A. Urlacher V. Buttner K. Lin H. Hecker M. (2010). Quality control of inclusion bodies in *Escherichia coli*. Microb. Cell Fact. 9, 41 10.1186/1475-2859-9-41 20509924 PMC2893105

[B27] Kudou M. Ejima D. Sato H. Yumioka R. Arakawa T. Tsumoto K. (2011a). Refolding single-chain antibody (scFv) using lauroyl-L-glutamate as a solubilization detergent and arginine as a refolding additive. Protein Expr. Purif. 77, 68–74 10.1016/j.pep.2010.12.007 21195184

[B28] Kudou M. Yumioka R. Ejima D. Arakawa T. Tsumoto K. (2011b). A novel protein refolding system using lauroyl-l-glutamate as a solubilizing detergent and arginine as a folding assisting agent. Protein Expr. Purif. 75, 46–54 10.1016/j.pep.2010.08.011 20817098

[B29] Lethanh H. Neubauer P. Hoffmann F. (2005). The small heat-shock proteins IbpA and IbpB reduce the stress load of recombinant *Escherichia coli* and delay degradation of inclusion bodies. Microb. Cell Fact. 4, 6 10.1186/1475-2859-4-6 15707488 PMC552319

[B30] Liovic M. Ozir M. Zavec A. B. Peternel S. Komel R. Zupancic T. (2012). Inclusion bodies as potential vehicles for recombinant protein delivery into epithelial cells. Microb. Cell Fact. 11, 67 10.1186/1475-2859-11-67 22624805 PMC3434093

[B58] Lindner A. B. Madden R. Demarez A. Stewart E. J. Taddei F. (2008). Asymmetric segregation of protein aggregates is associated with cellular aging and rejuvenation. Proc. Natl. Acad. Sci. U.S.A. 105, 3076–3081 10.1073/pnas.0708931105 18287048 PMC2268587

[B31] Maji S. K. Perrin M. H. Sawaya M. R. Jessberger S. Vadodaria K. Rissman R. A. (2009). Functional amyloids as natural storage of peptide hormones in pituitary secretory granules. Science 325, 328–332 10.1126/science.1173155 19541956 PMC2865899

[B32] Miller J. Arrasate M. Shaby B. A. Mitra S. Masliah E. Finkbeiner S. (2010). Quantitative relationships between huntingtin levels, polyglutamine length, inclusion body formation, and neuronal death provide novel insight into huntington's disease molecular pathogenesis. J. Neurosci. 30, 10541–10550 10.1523/JNEUROSCI.0146-10.2010 20685997 PMC3078518

[B33] Orr H. T. Zoghbi H. Y. (2007). Trinucleotide repeat disorders. Annu. Rev. Neurosci. 30, 575–621 10.1146/annurev.neuro.29.051605.113042 17417937

[B34] Peternel S. Jevsevar S. Bele M. Gaberc-Porekar V. Menart V. (2008). New properties of inclusion bodies with implications for biotechnology. Biotechnol. Appl. Biochem. 49, 239–246 10.1042/BA20070140 17708747

[B35] Radchenko E. Rogoza T. Khokhrina M. Drozdova P. Mironova L. (2011). SUP35 expression is enhanced in yeast containing [ISP+], a prion form of the transcriptional regulator Sfp1. Prion 5, 317–322 10.4161/pri.18426 22156729 PMC4012397

[B36] Rane A. M. Jonnalagadda S. Li Z. (2013). On-column refolding of bone morphogenetic protein-2 using cation exchange resin. Protein Expr. Purif. 90, 135–140 10.1016/j.pep.2013.05.008 23748143

[B37] Rinas U. Hoffmann F. Betiku E. Estape D. Marten S. (2007). Inclusion body anatomy and functioning of chaperone-mediated *in vivo* inclusion body disassembly during high-level recombinant protein production in *Escherichia coli*. J. Biotechnol. 127, 244–257 10.1016/j.jbiotec.2006.07.004 16945443

[B38] Rosano G. L. Ceccarelli E. A. (2009). Rare codon content affects the solubility of recombinant proteins in a codon bias-adjusted *Escherichia coli* strain. Microb. Cell Fact. 8, 41 10.1186/1475-2859-8-41 19630980 PMC2723077

[B39] Sabate R. De Groot N. S. Ventura S. (2010). Protein folding and aggregation in bacteria. Cell. Mol. Life Sci. 67, 2695–2715 10.1007/s00018-010-0344-4 20358253 PMC11115605

[B40] Sabate R. Espargaro A. Saupe S. J. Ventura S. (2009). Characterization of the amyloid bacterial inclusion bodies of the HET-s fungal prion. Microb. Cell Fact. 8, 56 10.1186/1475-2859-8-56 19863787 PMC2774669

[B41] Sabate R. Villar-Pique A. Espargaro A. Ventura S. (2012). Temperature dependence of the aggregation kinetics of Sup35 and Ure2p yeast prions. Biomacromolecules 13, 474–483 10.1021/bm201527m 22176525

[B42] Schrodel A. Volz J. De Marco A. (2005). Fusion tags and chaperone co-expression modulate both the solubility and the inclusion body features of the recombinant CLIPB14 serine protease. J. Biotechnol. 120, 2–10 10.1016/j.jbiotec.2005.04.028 16023240

[B43] Singh S. M. Sharma A. Upadhyay A. K. Singh A. Garg L. C. Panda A. K. (2012). Solubilization of inclusion body proteins using n-propanol and its refolding into bioactive form. Protein Expr. Purif. 81, 75–82 10.1016/j.pep.2011.09.004 21964443

[B44] Tsolis A. C. Papandreou N. C. Iconomidou V. A. Hamodrakas S. J. (2013). A consensus method for the prediction of 'aggregation-prone' peptides in globular proteins. PLoS ONE 8:e54175 10.1371/journal.pone.0054175 23326595 PMC3542318

[B45] Tyedmers J. Mogk A. Bukau B. (2010). Cellular strategies for controlling protein aggregation. Nat. Rev. Mol. Cell Biol. 11, 777–788 10.1038/nrm2993 20944667

[B46] Upadhyay A. K. Murmu A. Singh A. Panda A. K. (2012). Kinetics of inclusion body formation and its correlation with the characteristics of protein aggregates in *Escherichia coli*. PLoS ONE 7:e33951 10.1371/journal.pone.0033951 22479486 PMC3315509

[B47] Vazquez E. Corchero J. L. Burgueno J. F. Seras-Franzoso J. Kosoy A. Bosser R. (2012). Functional inclusion bodies produced in bacteria as naturally occurring nanopills for advanced cell therapies. Adv. Mater. 24, 1742–1747 10.1002/adma.201104330 22410789

[B48] Vera A. Aris A. Carrio M. Gonzalez-Montalban N. Villaverde A. (2005). Lon and ClpP proteases participate in the physiological disintegration of bacterial inclusion bodies. J. Biotechnol. 119, 163–171 10.1016/j.jbiotec.2005.04.006 15967532

[B49] Villar-Pique A. Espargaro A. Sabate R. De Groot N. S. Ventura S. (2012). Using bacterial inclusion bodies to screen for amyloid aggregation inhibitors. Microb. Cell Fact. 11, 55 10.1186/1475-2859-11-55 22553999 PMC3495732

[B50] Villar-Pique A. Ventura S. (2012). Modeling amyloids in bacteria. Microb. Cell Fact. 11, 166 10.1186/1475-2859-11-166 23272903 PMC3539947

[B51] Villaverde A. Garcia-Fruitos E. Rinas U. Seras-Franzoso J. Kosoy A. Corchero J. L. (2012). Packaging protein drugs as bacterial inclusion bodies for therapeutic applications. Microb. Cell Fact. 11, 76 10.1186/1475-2859-11-76 22686540 PMC3538617

[B52] Walsh D. J. Noble G. P. Piro J. R. Supattapone S. (2012). Non-reducing alkaline solubilization and rapid on-column refolding of recombinant prion protein. Prep. Biochem. Biotechnol. 42, 77–86 10.1080/10826068.2011.564256 22239709 PMC3329176

[B53] Walther C. Mayer S. Trefilov A. Sekot G. Hahn R. Jungbauer A. (2014). Prediction of inclusion body solubilization from shaken to stirred reactors. Biotechnol. Bioeng. 111, 84–94 10.1002/bit.24998 23860724

[B54] Wang L. (2009). Towards revealing the structure of bacterial inclusion bodies. Prion 3, 139–145 10.4161/pri.3.3.9922 19806034 PMC2802778

[B55] Wasmer C. Benkemoun L. Sabate R. Steinmetz M. O. Coulary-Salin B. Wang L. (2009). Solid-state NMR spectroscopy reveals that E. coli inclusion bodies of HET-s(218-289) are amyloids. Angew. Chem. Int. Ed Engl. 48, 4858–4860 10.1002/anie.200806100 19472238

[B56] Winkler J. Seybert A. Konig L. Pruggnaller S. Haselmann U. Sourjik V. (2010). Quantitative and spatio-temporal features of protein aggregation in *Escherichia coli* and consequences on protein quality control and cellular ageing. EMBO J. 29, 910–923 10.1038/emboj.2009.412 20094032 PMC2837176

[B57] Zheng H. Miyakawa T. Sawano Y. Yamagoe S. Tanokura M. (2013). Expression, high-pressure refolding and purification of human leukocyte cell-derived chemotaxin 2 (LECT2). Protein Expr. Purif. 88, 221–229 10.1016/j.pep.2013.01.008 23337084

